# Digestive System Anatomy and Feeding Mechanism of *Quatuoralisia malakhovi* (Hemichordata, Torquaratoridae)

**DOI:** 10.1134/S0012496623600100

**Published:** 2023-12-19

**Authors:** V. V. Malakhov, A. I. Lukinykh, O. V. Ezhova

**Affiliations:** Moscow State University, Moscow, Russia

**Keywords:** acorn worms, deep-sea Enteropneusta, morphology, digestive tract, faecal cords, Bering Sea

## Abstract

The digestive system was anatomically studied in the deep-sea enteropneust *Quatuoralisia mala-khovi*. It was shown that lateral collar lips are twisted in such a way that they form a ciliary groove that leads to an internal channel, through which collected detritus particles are transferred to peripheral pharyngeal channels. The size of the selected particles ranges from 1–6 to 100–200 μm, which corresponds to feeding on the remains of planktonic diatoms. A fecal cord was observed to act as an anchor that holds the heavily watered jelly-like body of Torquaratoridae at the sea floor during feeding.

Starting from the 1960s, mysterious structures shaped as regular helices and sinusoids have been observed in images taken in various oceanic regions at bathyal to abyssal depth [1]. It was not until 2005 that the mysterious structures were identified as fecal cords of Torquaratoridae, a new family of deep-sea acorn worms [2]. Torquaratoridae have been found to be a species-abundant group of a worldwide distribution [3–7]. The biology of Torquaratoridae is poorly understood. Torquaratoridae have an epibenthic lifestyle in contrast to the burrowing shallow-water acorn worms. It remains unclear how Torquaratoridae select detritus particles from sediments. The objective of this work was to study the digestive system anatomy and feeding mechanism in *Quatuoralisia malakhovi* Ezhova et Lukinykh, 2022.

Material for the study was collected on June 18, 2018, during the 82nd cruise onboard the RV *Akademik M.A. Lavrentyev*. Trawling was performed at station LV 82-9 (55.3451–55.3466 N, 167.2750–167.2752 E) in the Komandorsky Graben (the Volcanologists Massif) of the Bering Sea at depths of 1957–1933 m. *Quatuoralisia malakhovi* specimens were collected using the remotely operated underwater vehicle *Comanche 18* and fixed with 8% formalin in seawater for histological examination. Specimens were washed to remove the fixative and dehydrated with increasing ethanol concentrations by standard methods. Fragments prepared for histological examination were embedded in Paraplast, and the resulting blocks were used to obtain series of 10-µm histological sections with a Leica RM 2125 microtome. The sections were stained with Carracci hematoxylin and eosin (an ethanol solution). Scanning electron microscopy (SEM) was additionally used to study certain structural details of the *Q. malakhovi* digestive system. Material for SEM was dehydrated with acetone by a standard method, subjected to critical point drying with CO_2_ (HCP-2 critical point dryer, Hitachi, 1980), coated with a gold/palladium mixture (EIKO IB-3 ion coater, 1980), and examined under a JSM-6380LA microscope (JEOL, 2005).

Like in all hemichordates, the *Q. malakhovi* body is divided into three parts: a proboscis, a collar, and a long trunk (Fig. 1). The external morphology of *Q. malakhovi* has been described in detail previously [8]. The mouth is between the proboscis and collar on the ventral side of *Q. malakhovi* (Fig. 2, *mo*). The collar has symmetrical ear-shaped outgrowths known as the lateroal collar lips (Figs. 1, 2; *lp*). Based on in situ intravital observations, *Q. malakhovi* has an epibenthic lifestyle and moves on its flattened ventral side over the sediment surface. The ends of the lateral collar lips are immersed in the upper sediment layer and leave distinct furrows (Fig. 1, *fs*). An ectodermal epithelium coats the lateral collar lips and the total *Q. malakhovi* body and contains numerous unicellular mucous glands [8]. The lateral collar lips are twisted in such a way that they form a ciliary groove that goes along the anterior ventral margin of the lip (Fig. 2, *cg*) and leads to an internal channel of the lip (Fig. 2, *icl*). A characteristic mushroom-like pattern is formed in cross-sections of the collar lip of the fixed worms by the ciliary groove and the internal channel (Fig. 2, *1*). The width of the ciliary groove varies from 0 to 200 µm among sections.

**Fig. 1.**
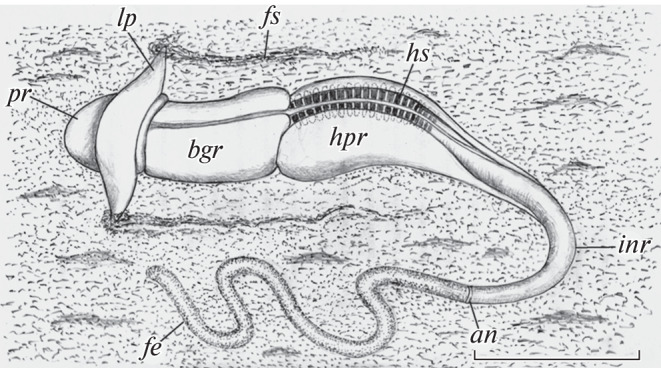
Feeding *Q. malakhovi* on the sediment surface; the image is based on underwater photographs. Bar, 5 cm. Designations: *an,* anus; *bgr,* branchiogenital subregion of the trunk; *fe,* fecal cord; *fs,* furrows left in the sediment by the lateral collar lips; *hpr,* hepatic subregion of the trunk; *hs,* hepatic sacculations; *inr,* intestinal subregion of the trunk; *lp,* lateral collar lips; *pr,* proboscis.

**Fig. 2.**
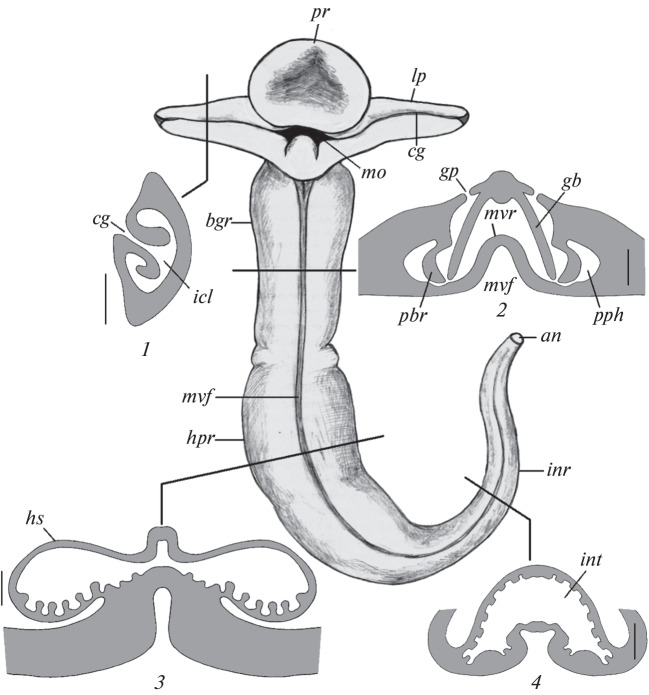
The external view of *Q. malakhovi* from the ventral side and diagrams of the transverse sections at the respective levels: *1,* through the lateral collar lip; *2,* through the branchiogenital subregion of the trunk; *3,* through the hepatic subregion of the trunk; and *4*, through the intestinal subregion of the trunk. Bar, 1 mm. Designations: *cg,* ciliary groove on the lateral collar lip; *gb,* gill bars; *gp,* gill pores; *icl,* internal channel within the lateral collar lip; *int,* intestine; *mo,* mouth; *mvf,* midventral furrow; *mvr,* midventral ridge; *pbr,* parabranchial ridges; *pph*, peripheral pharyngeal channels. The other designations are as in Fig. 1.

The *Q. malakhovi* trunk consists of three subregions: branchiogenital, hepatic, and intestinal (Fig. 2). The branchiogenital subregion harbors symmetrical rows of gill pores, which are hided under lateral wings folded onto the dorsal side. A gill pharynx corresponds to the branchiogenital subregion in the digestive tract (Fig. 2, *2*). There is no buccal cavity in *Q. malakhovi*, and the mouth leads directly into the gill pharynx. A midventral furrow runs on the ventral side along the total *Q. malakhovi* trunk (Fig. 2, *mvf*). The midventral furrow sinks deep into the trunk, and a midventral ridge consequently runs along the total digestive tract to divide the pharynx and subsequent regions of the digestive tract into the left and right halves (Fig. 2, *mvr*). Two symmetrical rows of gill slits separated by gill bars run along the dorsal side of the gill pharynx. Primary gill bars form dorso-lateral walls of the gill pharynx, and secondary gill bars dangle in the cavity of the gill pharynx and almost reach its ventral wall (Fig. 2, *2*). Parabranchial ridges (Fig. 2, *pbr*) divide the pharyngeal cavity into a central zone and two symmetrical peripheral pharyngeal channels (Fig. 2, *2*). The epithelium of the lower edge of the parabranchial ridges and the gastrodermis of the bottom of the pharynx are close together and contact each other in some sections, so that the peripheral pharyngeal channels are partly isolated from the central zone of the pharynx. The parabranchial ridges decrease in height in the posterior part of the pharynx, and the peripheral pharyngeal channels merge to form a short esophagus, which is continuous with the hepatic part of the digestive tract.

The hepatic subregion of the trunk is sharply separated from the branchiogenital subregion by a transversal groove running on the surface of the lateral wings (Fig. 1). The hepatic part of the digestive tract corresponds to the hepatic subregion. Brown-green metameric hepatic sacculations are on the dorsal side of the hepatic subregion (Fig. 1, *hs*). Symmetrical diverticula of the gut are inserted into the hepatic sacculations. The gastrodermis forms multiple folds on the ventral walls of these diverticula (Fig. 2, *3*).

The hepatic part of the digestive tract is continuous with its intestinal part without a marked boundary (Fig. 2, *4*). The intestinal wall is plicate (Fig. 2, *int*) and can stretch to a great extent. Fecal masses usually fill the intestinal cavity and are continuous with a dense fecal cord, which stretches behind the animal crawling on the sea floor (Fig. 1,  *fe*).

The majority of Enteropneusta are burrowing animals. Many of them eat mud and digest detritus and small organisms contained in it. Other species have a long proboscis, which is protruded from a burrow to collect detritus particles via a mucociliary mechanism. A dorsal groove occurs on the proboscis in these species to transport detritus particles to the base of the proboscis and then to the mouth with the use of a U-shaped ciliated organ [9–11]. In Torquaratoridae, the proboscis is small and the ciliated organ absent. However, the lateral collar lips are well developed and act as a main organ to sustain suspension feeding. In *Q. malakhovi*, the lateral lips are folded so that a ciliary groove goes on their ventral surface, which faces the sediment, and leads into a ciliated internal channel. Similar grooves are found in lateral lips of other Torquaratoridae species [2–5]. The lips of a crawling animal turn up the surface sediment layer, and small sediment particles are moved into the ciliary groove by the beating of cilia of the ectodermal epithelium of the lips and are transported to the mouth through the ciliated internal channel. The lateral lip structure has been studied in histological sections only in *Yoda demiankoopi* [5] apart from *Q. malakhovi.* Judging from sections, the width of the ciliary groove used to draw detritus particles into the internal channel of the collar lip does not exceed 200 µm in *Q. malakhovi* (see above). The width is similarly of about 200 µm in *Y. demiankoopi* [5]. The width determines the upper size limit of selectable detritus particles.

Species of Harrimaniidae and Ptychoderidae have symmetrical parabranchial ridges in the pharynx. The ridges lie in the parafrontal plane and divide the pharynx into dorsal respiratory and ventral digestive parts [12–14]. In *Q. malakhovi* and other Torquaratoridae, the pharynx is flattened in the frontal plane and the parabranchial ridges lie in the parasagittal plane, so that two lateral channels (the peripheral pharyngeal channels) form in the pharynx in place of a single ventral channel [3–5]. It is possible to think that sediment particles collected by the lateral collar lips are transported directly into the peripheral pharyngeal channels.

Cilia of the gill bars ensure filtration of the water that flows out the gill pores. In *Balanoglossus gigas*, particles larger than 1–2 µm cannot pass through the gill pores in *Balanoglossus gigas* [11]. In *Harrimania planktophilus,* the upper size limit of particles freely passing through the gill pores is 5.8 µm [15]. The data make it possible to assume that particles selected by Torquaratoridae range in size from 1–6 to 100–200 µm. Remains of planktonic diatoms of the genera *Thalassiosira, Coscinodiscus, Actinocyclus, Chaetoceros,* and others have predominantly been observed in *Q. malakhovi* intestinal contents and fall within the above range [16].

Hepatic sacculations are formed by the dorsal gastrodermis of the hepatic part in many species of Spengelidae, Ptychoderidae, and Torquaratoridae [13, 14]. Numerous digestive vacuoles are found in the gastrodermis of the hepatic subregion, suggesting intense intracellular digestion [17–19].

The mucus-bonded dense fecal cord is not degraded after leaving the anus in Torquaratoridae. A helical or intricately coiled fecal cord may be several times longer than the acorn worm as underwater photographs have shown [1–6]. When the intestine is completely empty, torquaratorid acorn worms can rise from the sea floor and move with underwater flows [4, 6, 20]. Hence, the fecal cord acts as an anchor that holds the heavily watered jelly-like body of Torquaratoridae at the sea floor during feeding.
